# High Stability, Piezoelectric Response, and Promising Photocatalytic Activity on the New Pentagonal CGeP_4_ Monolayer

**DOI:** 10.1021/acsphyschemau.4c00068

**Published:** 2024-12-04

**Authors:** José A. S. Laranjeira, Nicolas Martins, Pablo A. Denis, Julio Sambrano

**Affiliations:** † Modeling and Molecular Simulation Group, São Paulo State University (UNESP), School of Sciences, Bauru 17033-360, Brazil; ‡ Computational Nanotechnology, DETEMA, Facultad de Química, UDELAR, CC 1157, Montevideo 11800, Uruguay

**Keywords:** penta-graphene, piezoelectricity, CGeP_4_, graphene, 2D material

## Abstract

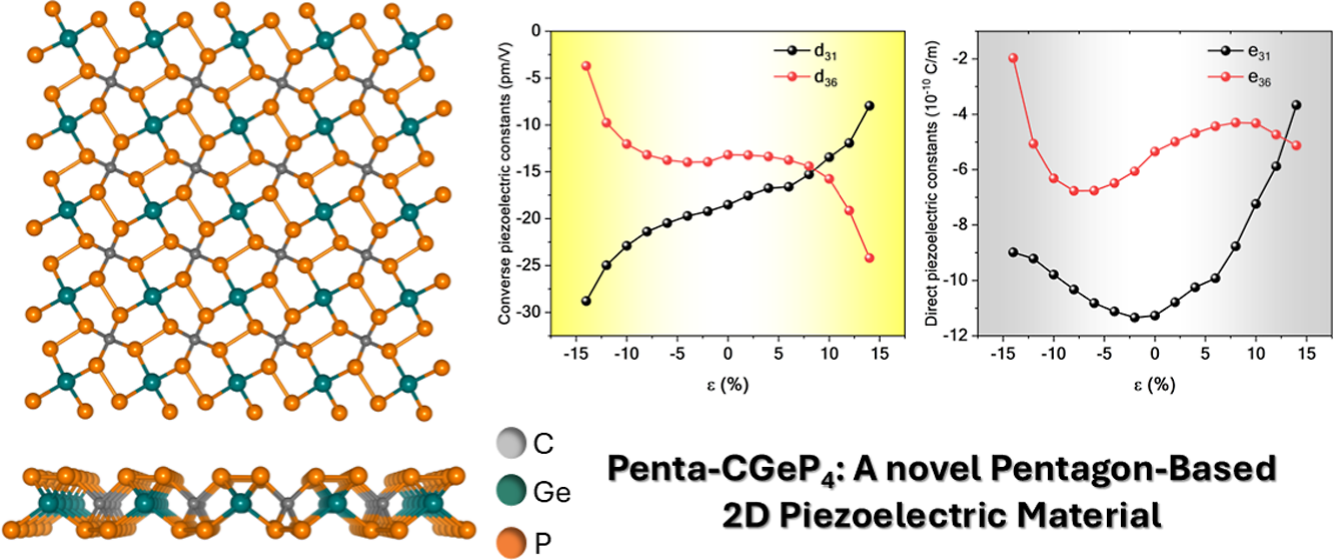

This study introduces the penta-structured semiconductor p-CGeP_4_ through density functional theory simulations, which possesses an indirect band gap transition of 3.20 eV. Mechanical analysis confirms the mechanical stability of p-CGeP_4_, satisfying Born–Huang criteria. Notably, p-CGeP_4_ has significant direct (*e*_31_ = −11.27 and *e*_36_ = −5.34 × 10^–10^ C/m) and converse (*d*_31_ = −18.52 and *d*_36_ = −13.18 pm/V) piezoelectric coefficients, surpassing other pentagon-based structures. Under tensile strain, the band gap energy increases to 3.31 eV at 4% strain, then decreases smoothly to 1.97 eV at maximum stretching, representing an ∼38% variation. Under compressive strain, the band gap decreases almost linearly to 2.65 eV at −8% strain and then drops sharply to 0.97 eV, an ∼69% variation. Strongly basic conditions result in a promising band alignment for the new p-CGeP_4_ monolayer. This suggests potential photocatalytic behavior across all tensile strain regimes and significant compression levels (ε = 0% to −8%). This study highlights the potential of p-CGeP_4_ for groundbreaking applications in nanoelectronic devices and materials engineering.

## 1 Introduction

The prediction of penta-graphene, also called p-CCC, in 2015 has added a new member to the extensive family of promising two-dimensional (2D) carbon allotropes.^1^ p-CCC has an outstanding negative Poisson’s ratio (NPR), which characterizes it as an auxetic nanomaterial with piezoelectricity and interesting optical properties.^2^ The Cairo-type tessellation recognizes the building blocks of p-CCC with two out-plane distorted pentagons, which breaks the π-conjugation in this 2D material.^3^ Studies suggest that the p-CCC structure can be obtained by employing chemical exfoliation using T12-carbon via dehydrogenation mechanism.^4^ Additionally, the isolation of C_20_ fullerene also helps the synthesis availability of p-CCC in view of its composition by 12 pentagonal rings inside the cage.^5^

The intriguing pattern of p-CCC has motivated the research on pentagonal-based inorganic structures. In this sense, unitary pentagonal-based sheets such as penta-silicene and penta-germanene emerged with significant ferroelectricity and low thermal conductivity.^6^ Extending for binary pentagon-based structures, p-CN_2_,^7^ p-BC_2_,^8^ p-SiC_2_,^9^ p-NiN_2_,^3^ and p-PdSe_2_^10^ are examples of stable compounds. Qu et al.^11^ also evaluated the intrinsic photocatalytic application of 9 transition-metal MX_2_ (M = Ni, Pd, and Pt, and X = S, Se, and Te) pentagon-based monolayers motivated by the synthesis of p-PdSe_2_, which was obtained by micromechanical exfoliation of the bulk crystals.^10^

Recently, the proposition of ternary 2D pentagon-based structures has aroused the interest of many researchers, resulting in new applications and distinct properties.^12^ For instance, p-BCN has intrinsic piezoelectricity and spontaneous polarization, which is significantly superior to the hexagonal boron nitride (h-BN) sheet.^13^ Furthermore, the p-BNSi predicted by Varjovi and colleagues^14^ possesses a high level of exciton binding and strong light absorption in the visible region. A semiconductor behavior is verified in the recently proposed p-SiXY_4_ (X = Si, C, Ge; Y = C, B, N), which reveals suitable band edge alignment for photocatalytic water splitting.^15^ The NPR observed in p-CCC also emerges for the p-SiCN structure, whose monolayer is tunable under strain in terms of geometry (buckled-to-planar transition) and mechanical properties.^16^

Strain engineering has proven to be an effective strategy for tuning the electronic and mechanical properties of 2D pentagon-based materials. Applying biaxial strain on the p-BCN, Dabsamut and co-workers^17^ have found a narrow band gap variation from 1.77 to 1.36 eV. Guo and Wang unveiled that^18^ a compressive biaxial strain of 2% can slightly increase the piezoelectric coefficients of p-CCC by 3.1%. Liu et al.^19^ showed that a small uniaxial strain to the p-BP_5_ monolayer can shift its band gap from quasi-direct to direct, while a moderate biaxial strain can transform the p-BP_5_ into a metal. In-plane tensile or compressive strain can modulate the carrier transport on p-SiC_2_.^20^ A uniaxial compressive strain of −8% along the *a*-direction significantly boosts the hole mobility along the *b*-direction by nearly 3 orders of magnitude, reaching 1.14 × 10^6^ cm^2^ V^–1^ s^–1^.

In view of the above-mentioned, this work aims to introduce p-CGeP_4_ via density functional theory (DFT) simulations. A detailed study of its electronic, structural, mechanical, and vibrational properties was performed to characterize this new structure. Considering the potentiality of strain engineering for modulating the properties of 2D structures, the effects of biaxial strain on the electronic and piezoelectric properties of p-CGeP_4_ were studied. It is expected that this study can highlight the promise of p-CGeP_4_ for groundbreaking applications in nanoelectronic devices and materials engineering.

## 2 Computational Methodology

Computational simulations were carried out using the CRYSTAL17 package^21^ and based on the DFT in conjunction with the WC1LYP hybrid functional, which is described by the following equation: *E*_XC_[ρ] = *aE*_HF_^X^ + (1 – *a*)*E*_DFT_^X^ + *E*_DFT_^C^, where *E*_HF_^X^ is the exchange Hartree–Fock (HF) functional, *E*_DFT_^X^ is the WC exchange functional, *E*_DFT_^C^ denotes the LYP correlation functional, and *a* represents the mixing of the nonlocal HF exchange with the WC exchange functional (*a* = 16%). The triple-ζ valence with polarization^22^ basis set was used to describe the C, Ge, and P atomic centers. The same methodology was used in previous work.^9^

The precision of the infinite Coulomb and HF exchange series is controlled by five α_*i*_ parameters with *i* = 1, 2, 3, 4, and 5, where α_1_ is the overlap, α_2_ is the penetration for Coulomb integrals, α_3_ is the overlap for HF exchange integrals, and α_4_ and α_5_ are the pseudo-overlaps (HF exchange series). The two-electron contributions are neglected when the overlap between atomic functions is lower than . For the calculations, the five α_*i*_ parameters were set to 20, 20, 20, 20 and 40, respectively. The convergence criterion for the self-consistent field (SCF) is 10^–6^ au/cell, while for geometry optimization, it is 10^–7^ au/cell, and for elastic constant calculations, it is 10^–8^ au/cell. The optimization convergence was checked on the root-mean-square (RMS) and the absolute value of the largest component of both the gradients and estimated displacements. The convergence criteria employed in the optimization for RMS and the largest component for gradient were 0.00030 and 0.00045 au, and for displacement 0.00120 and 0.00180 au, respectively. The reciprocal space was sampled using Pack-Monkhorst and Gilat nets with sublattices and a shrinking factor of 8, resulting in 90 *k*-points in the irreductible Brillouin zone. The vibrational modes at the Γ point were evaluated using the numerical second derivatives of the total energies estimated with the coupled perturbed HF/Kohn–Sham algorithm.^23^

The quantum theory of atoms in molecules and crystals (QTAIMC)^24,25^ was employed to characterize the nature of chemical bonds. This approach uses the electronic density (ρ(*r*)) at the bond critical points (BCPs) to obtain topological parameters, such as the Laplacian (∇^2^ρ(*r*)), the potential energy density (*V*(*r*)), the kinetic energy density (*G*(*r*)), and the total electronic energy density (*H*(*r*) = *V*(*r*) + *G*(*r*)). These parameters can provide valuable information regarding the type of bond interaction: shared shell (covalent bonds) or closed electron (ionic bonds).

The elastic constants (*C*_*ij*_) were calculated as the second derivative of the energy (*E*) concerning the strain component (ϵ_*i*_ and ϵ_*j*_) according to the following expression
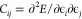
1

To analyze the anisotropic mechanical behavior of the structures presented herein, the orientation-dependent Young modulus *Y*(θ), Poisson ratio ν(θ), and shear modulus *G*(θ) were calculated employing the following expressions^26^
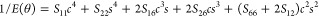
2
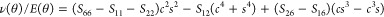
3
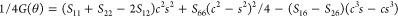
4where *s* = sin θ, *c* = cos θ, and θ ∈ [0, 2π] is the angle with respect to the +*x* axis. *S*_*ij*_ = *C*_*ij*_^–1^ is the elastic compliance constants.

The following equation calculated the direct (stress) piezoelectric constants , where |*e*| is the elementary charge, *a*_*ij*_ is the Cartesian component of the direct lattice basis vector, φ_*l*_ is the local function obtained utilizing the Berry phase approach, and η_v_ is the pure strain tensor.

Utilizing the Voigt notation for 2D materials and considering only in-plane strain components, the converse (strain) piezoelectric constant (*d*_*ij*_) was obtained through the following relation
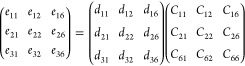
5

The thermodynamic stability of p-CGeP_4_ was assessed by computing its cohesive energy (*E*_coh_), which is defined by
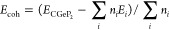
6where  is the total energy of the p-CGeP_4_ structure, *E*_*i*_ is the energy of an isolated atom *i* (C, Ge, P), and *n*_*i*_ is the number of *i* atoms in the sheet. By this definition, a negative value indicates an energetically stable material.

Molecular dynamics (MD) simulations were carried out using the extended tight-binding approximation (xTB)^27^ as implemented in the DFTB^+^ package,^28^ with the GFN1-xTB parametrization.^29^ The thermal stability of p-CGeP_4_ was studied using the Berendsen thermostat^30^ at 300 K by 40 ps with a time step of 1 fs. In the next step, MD simulations were conducted, starting at 300 K and continuing until structural rupture.

## 3 Results and Discussion

### 3.1 Structure and Stability

**Figure 1:**
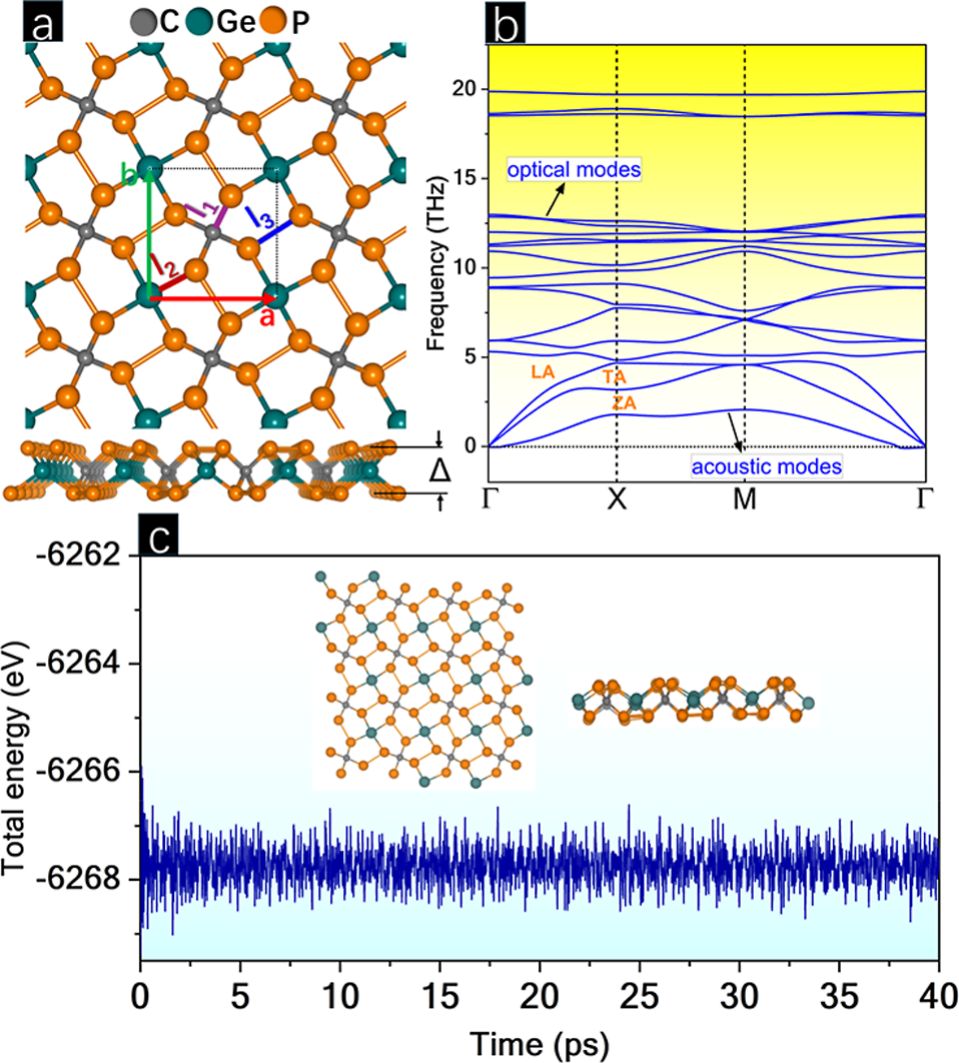
(a) p-CGeP_4_ lattice with unit cell and their three nonequivalent bonds highlighted, (b) phonon bands dispersion, and (c) MD simulation at 300 K of p-CGeP_4_ along with last iteration snapshot.

The novel pentagonal-based CGeP_4_ structure belongs to the space group *P*4̅ (no. 81) with a square symmetry, with lattice parameters *a* = *b* = 4.58 Å. As can be seen in Figure 1a, each unit cell of p-CGeP_4_ consists of carbon and germanium atoms, each 4-fold coordinated with phosphorus. The unit cell comprises three non-equivalent atoms with internal coordinates Ge (0.000, 0.000, 0.000), C (0.500, 0.500, 0.000), and P (0.634, 0.792, 0.064). The optimized p-CGeP_4_ has a buckling height (Δ) of 2.56 Å, superior to the p-CCC (Δ = 1.22 Å) and other ternary compounds like p-BCN (Δ = 1.37 Å) and p-CNP (Δ = 2.43 Å), all obtained at the DFT/WC1LYP level. In this pentagonal lattice, the *l*_1_ (C–P), *l*_2_ (Ge–P), and *l*_3_ (P–P) bond lengths are 1.95, 2.31, and 2.28 Å, respectively, values that support its higher thickness. The thermodynamic stability of p-CGeP_4_ was calculated via *E*_coh_, and a value of −4.17 eV/atom was obtained, which is comparable to those obtained for well-reported monolayers, such as p-CCC (−6.83 eV/atom), p-BCN (−6.38 eV/atom), and p-CNP (−5.12 eV/atom).

**Table 1:** Topological Parameters Based on the QTAIMC Analysis for *l*_1_, *l*_2_, and *l*_3_ Bonds in p-CGeP_4_, Where ρ(*r*) is the Charge Density, ∇^2^ρ(*r*) is the Laplacian of the Charge Density, |*V*(*r*)|/*G*(*r*) is the Ratio Between the Virial *V*(*r*) and the Kinetic Density Energy *G*(*r*), *H*(*r*)/ρ(*r*), and *G*/ρ(*r*)

bond	ρ(*r*)	∇^2^ρ(*r*)	|*V*(*r*)|/*G*(*r*)	*H*(*r*)/ρ(*r*)	*G*/ρ(*r*)
*l*_1_ (C–P)	0.128	–0.043	2.332	–0.340	0.340
*l*_2_ (Ge–P)	0.094	–0.111	3.154	–0.231	0.231
*l*_3_ (P–P)	0.104	–0.183	2.871	–0.408	0.408

The phonon bands dispersion is an important tool to certify the dynamic stability of 2D materials.^31^ Clearly, as presented in Figure 1b, the vibrational bands of p-CGeP_4_ do not have regions with imaginary frequencies, therefore indicating that p-CGeP_4_ is a free-standing monolayer. Additionally, the phonon dispersion curves of p-CGeP_4_ comprise 3 acoustic and 15 optical modes, which relate to the in-phase and out-phase vibrations, respectively. The acoustic branches can be attributed to the longitudinal acoustic mode (LA), transversal acoustic mode (TA) and out-of-plane acoustic mode (ZA), whose frequencies are 4.68, 4.58, and 2.06 THz around the *M* point, respectively, except for LA mode that occurs at *X* point. On the other hand, the highest optical mode occurs at 19.87 THz in the center of the Brillouin zone (Γ point). An absence of intersections or overlaps between the acoustic modes or between the acoustic and optical modes is noticed, indicating low phonon scattering channels. The thermal stability of the p-CGeP_4_ at room temperature (300 K) was verified by MD simulations, and the variation of the total energy as a function of the simulation time (40 ps) can be visualized in Figure 1c. Our dynamics suggest a small energy fluctuation and the absence of significant lattice distortion on the p-CGeP_4_ geometry, attesting to its feasibility. As detailed in the Supporting Information, the p-CGeP_4_ monolayer demonstrates impressive thermal stability, withstanding temperatures up to approximately 2050 K without experiencing ruptures or reconstructions.

### 3.2 Electronic Description

**Figure 2:**
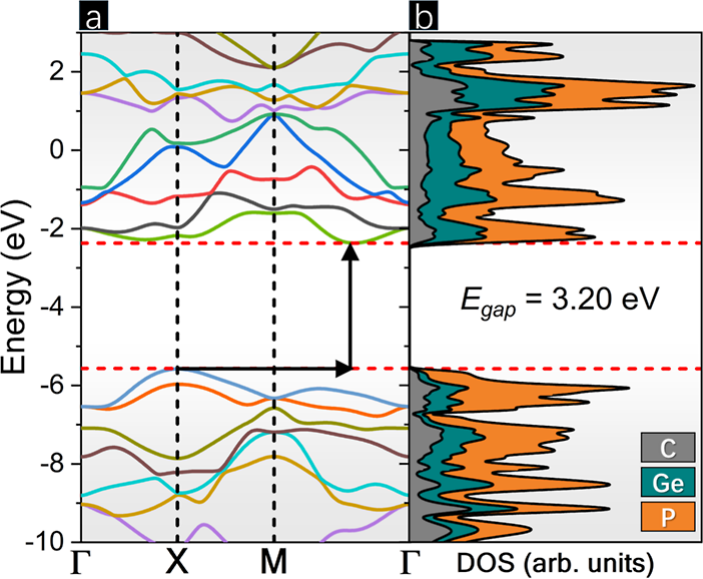
(a) Band structure and (b) density of states (DOS) for p-CGeP_4_.

The band structure and density of states (DOS) are listed in Figure 2. The band structure reveals an indirect band gap transition of 3.20 eV, with the valence band maximum (VBM) at the *X* point and the conduction band minimum (CBM) between the M and Γ points. The DOS shows a higher density of P states compared to Ge and C states along the valence and conduction bands, respectively. In the VBM, the DOS shows a softer behavior compared to the CBM, indicating higher band dispersion and, therefore, higher mobility of photogenerated holes. The similarity between the shapes of the DOS curves for all atoms reveals strongly correlated states in p-CGeP_4_.

The QTAIMC analysis was utilized to access the chemical character of the *l*_1_, *l*_2_, and *l*_3_ nonequivalent bonds of p-CGeP_4_. The electronic density topological parameters are listed in Table 1.

Espinosa et al.^32^ classify the bonds based on the |*V*(*r*)|/*G*(*r*) ratio at the BCPs. For closed-shell interactions, such as ionic bonds, we have |*V*(*r*)|/*G*(*r*) < 1, while open-shell interactions are expected to have |*V*(*r*)|/*G*(*r*) > 2. Values between 1 and 2 indicate a transitory region, where there is an early stage of covalent bond formation. Macchi et al.^33^ propose that *H*/ρ(*r*) > 0, and *G*/ρ(*r*) > 1 characterize closed-shell interactions. On the other hand, open-shell interactions are expected to have *H*/ρ(*r*) < 0, and *G*/ρ(*r*) < 1. As suggested by the DOS curves, the bonds are strongly covalent, i.e., possess higher closed-shell character due to its ∇^2^ρ(*r*) < 0, |*V*(*r*)|/*G*(*r*) > 2, and *H*/ρ(*r*) < 0. The *l*_3_ (P–P) and *l*_2_ (Ge–P) present the highest covalent character and |*V*(*r*)|/*G*(*r*) ratio, respectively.

### 3.3 Mechanical Properties

Table 2 shows the mechanical properties of p-CGeP_4_ and other pentagon-based monolayers. For square lattices, the Born–Huang criteria^34^ is described by *C*_11_ > 0, *C*_66_ > 0, and *C*_11_ > *|C*_12_*|*, which is fulfilled, confirming the mechanical stability of p-CGeP_4_. p-CGeP_4_ exhibits maximum and minimum Young modulus (*Y*_max_ and *Y*_min_) of 100.23 and 77.58 N/m, Poisson Ratio (ν_max_ and ν_min_) of 0.26 and 0.03, and shear modulus (*G*_max_ and *G*_min_) of 48.55 and 31.01 N/m, respectively. Notice that *C*_11_, *C*_22_, *C*_12_, *C*_66_, *G*_max_/*G*_min_, and *Y*_max_/*Y*_min_ are significantly lower than the values exhibited for the other penta-graphene-like structures. About the ν_max_/ν_min_, p-CGeP_4_ does not present a negative Poisson ratio as the other structures. Furthermore, p-CGeP_4_ has a remarkable anisotropy for this parameter, a feature not observed for the other monolayers. Differently from other ternary pentagon structures, p-CGeP_4_ has *C*_11_ = *C*_22_. This means that the combination of different species at the cation (sp^3^-site) results in a disorder lower than that in the anion site (sp^2^-site), which is corroborated by the fact that the p-CNP and p-BCP show rectangular lattices. At the same time, the p-CGeP_4_ remains with square symmetry as the p-CCC.

**Table 2:** Calculated Elastic Constants *C*_11_, *C*_12_, and *C*_66_ (N/m), Maximum and Minimum Values of Young Modulus (*Y*_max_/*Y*_min_) (N/m), Poisson Ratio (ν_max_/ν_min_), and Shear Modulus (*G*_max_/*G*_min_) (N/m) for p-CGeP_2_, and Other Pentagon-Based Monolayers Obtained at WC1LYP/DFT Level

	*C*_11_	*C*_22_	*C*_12_	*C*_66_	*Y*_max_/*Y*_min_	ν_max_/ν_min_	*G*_max_/*G*_min_
p-CGeP_4_	83.22	83.22	20.36	48.12	100.23/77.58	0.26/0.03	48.55/31.01
p-SiC_2_	151.52	151.52	6.26	82.46	161.27/151.27	0.04/–0.02	82.46/72.63
p-GeC_2_	136.50	136.50	7.65	72.72	144.80/136.07	0.08/–0.01	72.72/64.43
p-CNP	194.26	185.90	10.66	106.63	207.09/185.31	0.06/–0.03	106.63/89.67
p-BCN	187.21	229.18	7.46	114.51	230.11/186.97	0.03/–0.04	114.51/99.35
p-CCC	281.17	281.17	–22.49	160.39	286.40/279.38	–0.08/–0.11	160.39/151.83

**Figure 3:**
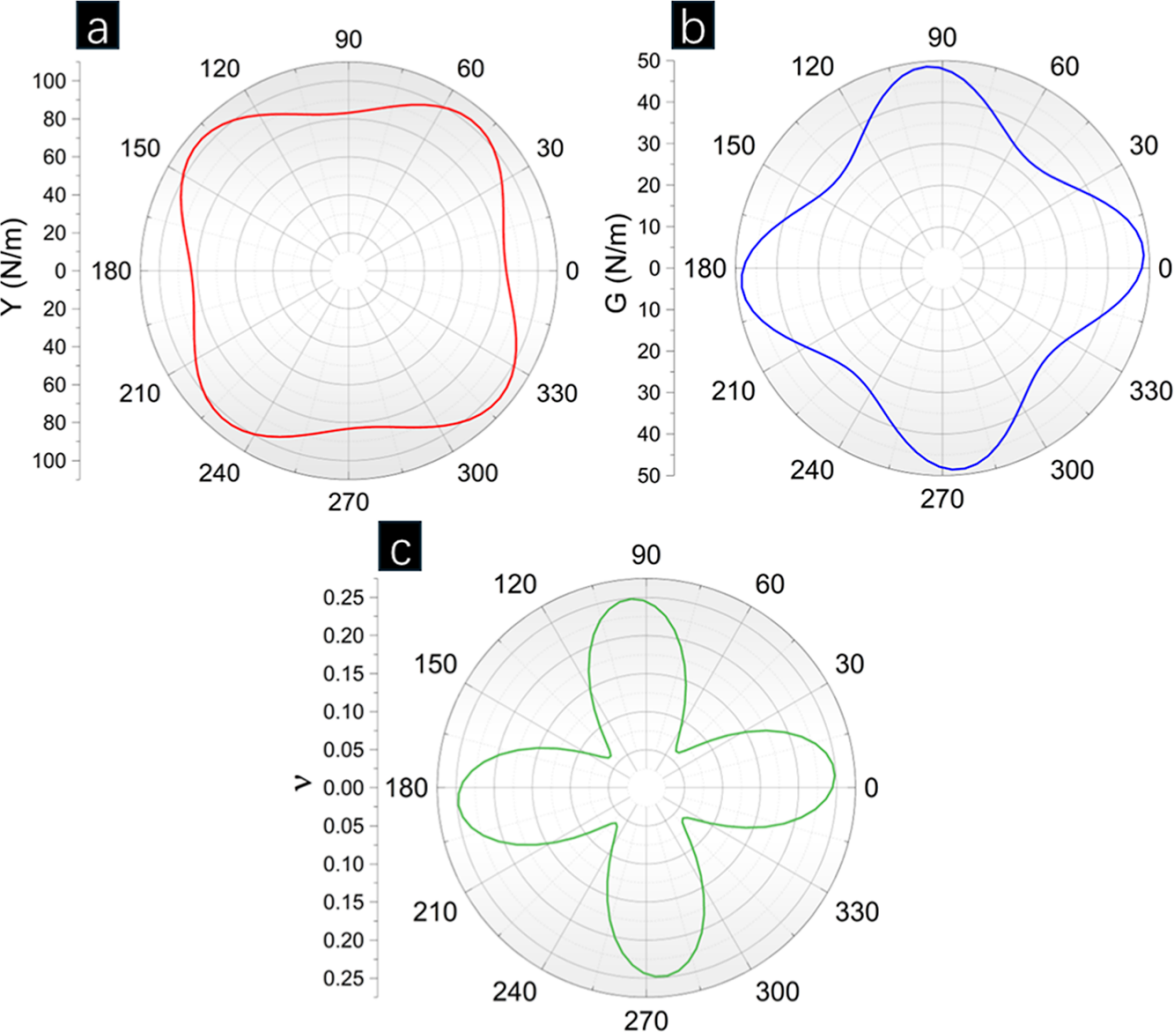
Representation of the angular dependence of (a) Young modulus (*Y*), (b) shear modulus (*G*), and (c) Poisson ratio (ν) for p-CGeP_4_.

The polar representations of Y, ν, and G were adopted to analyze the anisotropy for p-CGeP_4,_ as represented in Figure 3. It can be noticed that the anisotropy is more remarkable for ν. A distinctive characteristic of p-CGeP_4_ is their closer to zero ν at approximately *k*π/4 (*k* as an integer) angles, which indicates that for this orientation, the material does not change in lateral dimensions when stretched or compressed. The Y and G show a square-like shape with maximum values for angles closer to *k*π/3 and *k*π/4, respectively.

Due to their asymmetric crystalline structure, some materials can exhibit piezoelectricity, which allows them to generate polarization in response to mechanical stress or strain. The P-CGeP_4_ lattice only comprises the identity and three 2-fold rotation axes, not presenting an inversion center; i.e., it is a non-centrosymmetric structure, which gives it the ability to exhibit piezoelectricity.^35^ In view of this, the piezoelectric stress (*e*_*ij*_) and strain (*d*_*ij*_) constants of p-CGeP_4_ and other pentagon-based monolayers^9,17,18,36,37^ are represented in Table 3. It can be noticed that p-CGeP_4_ exhibits non-null direct (*e*_31_ = −11.27 and *e*_36_ = −5.34 × 10^–10^ C/m) and converse (*d*_31_ = −18.52 and *d*_36_ = −13.18 pm/V) piezoelectric coefficients, which are significantly higher than those obtained at the theory level for another pentagon-based structure, which makes this compound promising for advanced applications such as sensors, actuators, energy harvesting, and consumer electronics.

**Table 3:** Piezoelectric Stress (*e*_*ij*_) (10^–10^ C/m) and Strain (*d*_*ij*_) (pm/V) Constants of p-CGeP_4_ Monolayer Compared to Other Pentagonal-Based Structures (p-SiC_2_, p-GeC_2_, p-CNP, p-BCN, and p-CCC). All results were obtained at DFT/WC1LYP level.

system	*e*_11_	*e*_12_	*e*_31_	*e*_36_	*d*_11_	*d*_12_	*d*_31_	*d*_36_
p-CGeP_4_			–11.27	–5.34			–18.52	–13.18
p-SiC_2_				–0.61				–0.74
p-GeC_2_				–1.59				–2.20
p-CNP	–2.37	–0.44		–0.73	–1.22	–0.17		–0.69
p-BCN	–2.43	–1.40		1.29	–1.28	–0.56		1.13
p-CCC				–0.03				–0.02

### 3.4 Vibrational Analysis

**Figure 4:**
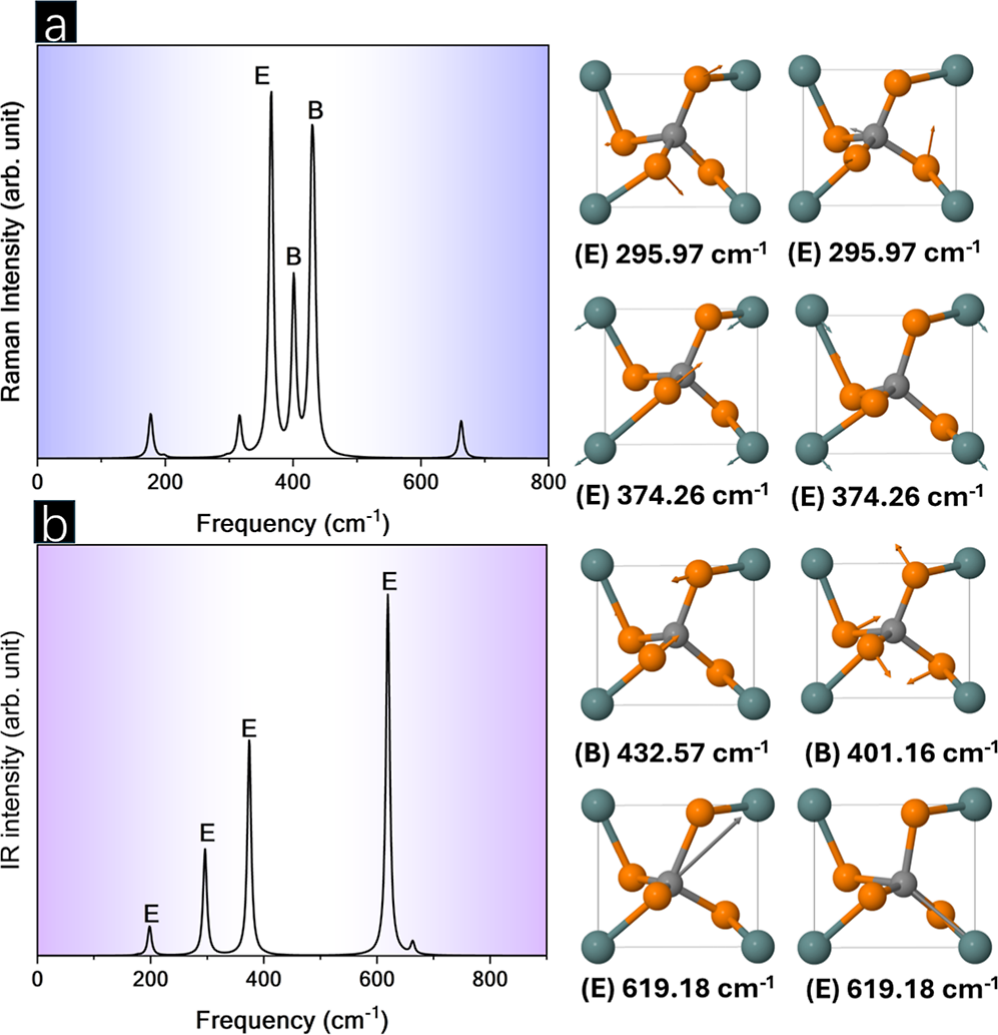
(a) Raman and (b) IR spectra along with the vibration associated with most intense peaks for both.

Vibrational analysis was utilized to analyze the short-range order in p-CGeP_4_. The Raman and IR spectra are shown in Figure 4. The presence of 18 active modes Γ_vib_ = 3*A* + 5*B* + 10*E* was verified to be all Raman-active and Γ_IR_ = *A* + 5*B* + 10*E* to be IR-active. Three main peaks observed in the Raman spectrum were at 374.26, 401.16, and 432.57 cm^–1^, with *E*, *B*, and *B* symmetries, respectively. At 374.26 cm^–1^, two doubly degenerate modes were noted, and the symmetric stretching of Ge–P bonds characterized the motion. For 401.16 cm^–1^, scissoring was noted, with the motion occurring due to the displacement of P atoms. In 432.57 cm^–1^, asymmetrical stretching was observed for the C–P and Ge–P bonds. Regarding the IR spectrum, the three most intense peaks occurred at 295.97, 374.26, and 619.18 cm^–1^, all being E doubly degenerate modes, i.e., each possessing two associated vibrations. At 295.97 and 619.18 cm^–1^, the vibrations were associated with the asymmetric stretching of the C–P bonds.

### 3.5 Strain Engineering

**Figure 5:**
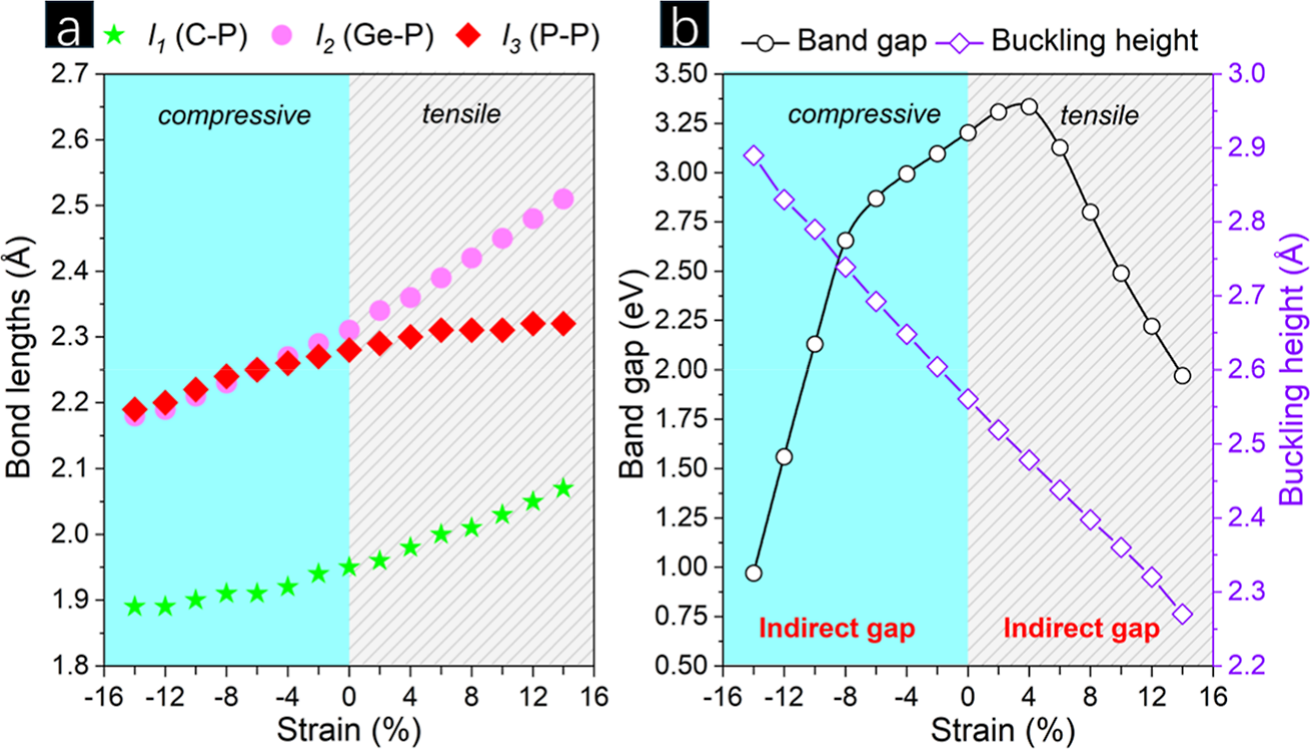
(a) Bond lengths and (b) band gap energy and buckling as a function of biaxial strain.

Here, the homogeneous biaxial strain (ε) is applied on the p-CGeP_4_ in both compressive and tensile regimes, following the relation ε = (*a* – *a*_0_)/*a*_0_, where the strained and unstrained lattice parameters are represented by *a* and *a*_0_, respectively. Our simulations suggest p-CGeP_4_ stability under a −14% to +14% range of biaxial strain. As a first analysis, the changes in the bond lengths induced by the strain deformation were assessed, as seen in Figure 5a. Under tensile strain, the out-of-plane bond lengths *l*_1_ (C–P) and *l*_2_ (Ge–P) increase, changing from 1.96 to 2.07 Å (*l*_1_) and 2.31 to 2.51 Å (*l*_2_) at maximum stretching (ε = +14%). Meanwhile, a small effect of this positive strain on the in-plane *l*_3_ (P–P) bond length is registered, and the 2.31 Å value found at ε = +6% is maintained until +14% of strain, which is closer to that noticed in the strain-free state (2.28 Å). Thus, this behavior suggests a minor push strength effect on the P–P bond. On the other hand, the compressive regime acts noticeably in the Ge–P bond, modifying it to 2.18 Å (ε = −14%), being slightly smaller than those found at the same level of compression for P–P bond length (2.19 Å). Therefore, the maximum compression rearranges the bond length order observed at equilibrium (ε = 0%), i.e., *l*_2_ > *l*_3_ > *l*_1_ to *l*_3_ > *l*_2_ > *l*_1_.

The trend mentioned above can be related to the strain effect on the buckling height of p-CGeP_4_, which was plotted in Figure 5b. As the tension increases, the Δ parameter of 2.56 Å is mitigated to 2.28 Å when ε = +14% is applied, which favors the increment on the out-of-plane bonds, C–P and Ge–P. Oppositely, the compression induces a thickness enlargement, and Δ = 2.89 Å is found for ε = −14%. Our results met those reported for other pentagonal-based lattices, such as B_2_C,^8^ SiCN,^16^ and PBN,^38^ revealing stretching as a crucial factor in reducing the monolayer thickness.

In this work, the band gap variation of p-CGeP_4_ in the function of the biaxial strain is also analyzed, as noted in Figure 5b. Under the tensile regime, the *E*_gap_ grows up to 3.31 eV at 4% strain and then is smoothly reduced at the value of *E*_gap_ = 1.97 eV for maximum stretching, representing an ∼38% decrease compared to the band gap at equilibrium (*E*_gap_ = 3.20 eV). On the other hand, the negative strain decreases the band gap almost regularly until ε = −8%, with *E*_gap_ = 2.65 eV, whose value drops significantly to 0.97 eV at the maximum level of compression and denotes a variation of ∼69%. Using the HSE06 level, Li and co-workers^8^ appoint that p-B_2_C, *E*_gap_ = 2.47 eV, has its band gap decreased to 2.21 (1.83 eV) when 10% of compressive (tensile) strain is applied. The same trend occurs for p-PtSiTe, where the band gap of 0.80 eV (strain-free state) is tuned to ∼0.30 and 0.65 eV at ε = −10 (10%).^39^ P-BP_5_,^38^ p-Sb_2_Si,^40^ and P_2_C^41^ are some pentagonal sheets that present a similar band gap reduction by strain effect.

In general, in semiconductors with distinct ring circuits, such as tetragonal BN,^42^ SiC,^43^ and InN-based^44^ graphenylenes or GeC^45^ and ZnS^46^ honeycomb lattices, the compressive regime increases the band gap of the material. Here, both negative and positive deformations lead the p-CGeP_4_ band gap to the visible-light range, a required condition for many semiconductor devices.

**Figure 6:**
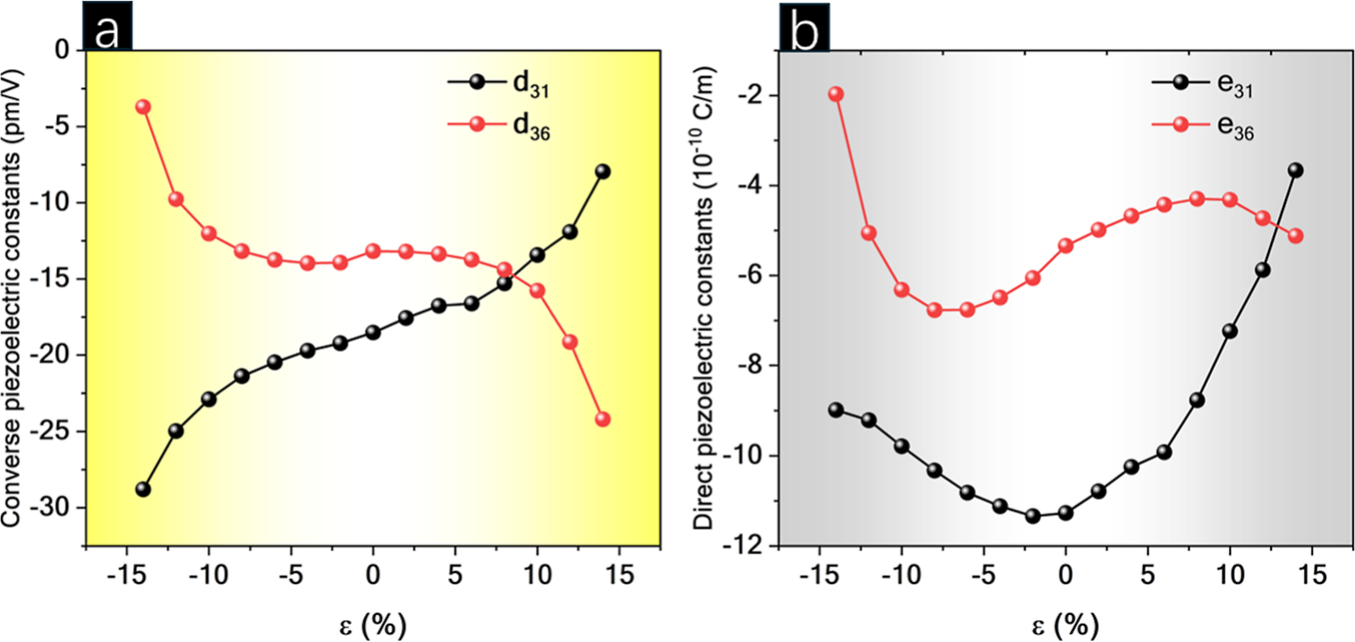
(a) Direct *e*_31_ and *e*_36_ and (b) converse *d*_31_ and *d*_36_ piezoelectric coefficients as a function of biaxial strain.

To take into account the piezoelectric response of p-CGeP_4_ when affected by biaxial strain, Figure 6 denotes the variation of the *e*_31_, *e*_36_, *d*_31_, and *d*_36_ coefficients. Both *e*_31_ and *e*_36_ coefficients decrease (in module) by tensile strain application and are equal to 3.66 and 5.12 × 10^–10^ C/m at maximum stretching, respectively (see Figure 6b). The same reduction occurs in negative strain for the *e*_31_ coefficient, whose value is found to be 8.69 × 10^–10^ C/m at maximum compression. On the contrary, the compression increases |*e*_36_| to 6.77 × 10^–10^ C/m when ε = −8% and then falls to 1.97 × 10^–10^ C/m (when ε = −14%). In addition to that, each of *d*_31_ and *d*_36_ components assume a distinct plot, as seen in Figure 6a. While |*d*_31_| increases from a strain-free state to the top level of compression, reaching 28.80 pm/V. The same coefficient becomes smaller as the stretching expands, and 7.95 pm/V is found. On the other hand, the |*d*_36_| of p-CGeP_4_ is enhanced when tensile strain is applied, 24.21 pm/V at ε = 14%, and reduced under the compressive regime, 3.71 pm/V at ε = −14%. Investigating the g-C_3_N_4_ monolayer, Guo and colleagues^47^ appoint a significant increase of 330% in the *d*_11_ coefficient comparing 4% of stretching with the equilibrium. Different to that reported here, Janus monolayers such as SbTeI and BiTeI do not have a strain-dependent *d*_31_ behavior, which suggests the huge importance of symmetry in this kind of property.^48^ In general, the piezoelectric properties of p-CGeP_4_ can be tuned by applying a selective strain range, and all coefficients maintain great values. As the p-CGeP_4_ is still a semiconductor when deformed (see Figure 5b), it can perform a promising role as a novel piezoelectric 2D material under mechanical strain.

**Figure 7:**
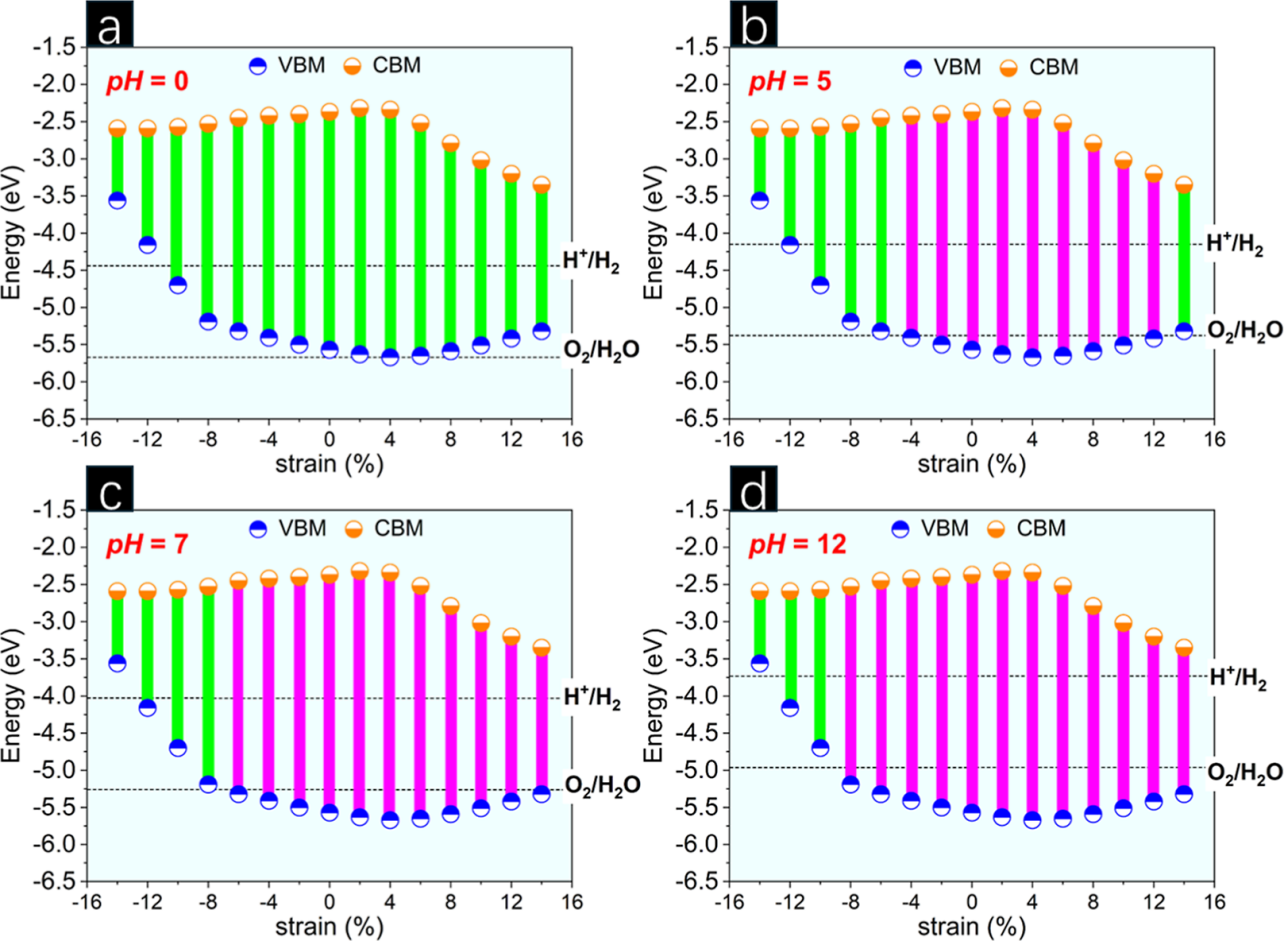
Band alignments of CGeP_4_ monolayer under biaxial strain at pH equal to (a) 0, (b) 5, (c) 7, and (d) 12.

Among the many strategies to make a material suitable for photocatalytic processes, strain engineering is one of the most promising solutions. As expected, the semiconductor should have an appropriate band gap for a visible-light-driven photocatalyst (1.5 < *E*_gap_ < 3.0 eV).^49^ By applying biaxial strain, this requirement can be achieved in p-CGeP_4_, as seen in Figure 7b, where the band gap reaches the visible range of the spectrum (390–760 nm) to harvest solar power. Another favorable condition for the potential use of p-CGeP_4_ as a photocatalyst is due to their indirect band gap feature since the electron–hole recombination is mitigated by the presence of distinct *K*-points between the VBM and CBM.^50^ The principle of water splitting consists of the photoexcited electron–hole pair separation that is driven to the surface without any recombination. Then, the photogenerated electrons (*e*^–^) reduce the water molecules into H_2_: 2H^+^ + 2*e*^–^ → H_2_, and in the same way, the induced holes (*h*^+^) oxidize H_2_O into O_2_: H_2_O^+^2*h*^+^ → 1/2O_2_ + 2H^+^.^51^ Aiming at water splitting purposes, the CBM position should be more positive than the reduction potential (H^+^/H_2_), −4.44 eV at pH = 0, and the VBM position should be more negative than the oxidation potential (O_2_/H_2_O), −5.67 eV at pH = 0.^52^

Here, the band alignment of p-CGeP_4_ is assessed under the biaxial strain effect and considering different pH conditions by using the equations^53^
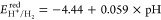
7
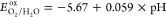
8

In the vacuum (pH = 0), the unstrained and deformed p-CGeP_4_ monolayer matches the H^+^/H_2_ potential (see Figure 7a). On the other hand, the VBM position of p-CGeP_4_ under all strain applications, even in a strain-free state, appears above the O_2_/H_2_O potential, which does not meet the full requirements for water splitting. A usual strategy to tune the water redox potentials of a material to attend photocatalytic promises can be applying an external bias potential by changing the pH environment.^54^ Therefore, Figure 7b–d denotes the band offsets when the photocatalytic surface is submitted to weakly acidic (pH = 5), neutral (pH = 7), and strongly basic conditions (pH = 12). Clearly, we can see a suitable band alignment at the acid environment for p-CGeP_4_ at equilibrium, as well as at most levels of tension (ε = 0% to +12%) and determined values (ε = −2% and −4%) of compression. The p-CGeP_4_ monolayer also sandwiches both H^+^/H_2_ and O_2_/H_2_*O* potentials for a neutral pH. Under pH = 7, the VBM position is also suitable for ε = +14% and ε = −6%, in addition to the appropriate region of strain mentioned in the acidic case. The strongly basic condition offers the most promising band alignment for the new p-CGeP_4_ monolayer, making it a pentagonal structure with potential photocatalyst behavior for all tensile strain regimes and also under significant levels of compression (ε = 0% to −8%). Thus, considering strongly acidic and basic pH values, and also the neutral pH, the p-CGeP_4_ substrates can promote photogenerated electrons and holes to drive the water-splitting reactions and could be useful as visible-light photocatalytic devices due to their suitable band gap under strain engineering.

## 4 Conclusions

A novel pentagonal CGeP_4_ monolayer (p-CGeP_4_) was unveiled, and its structural, electronic, vibrational, piezoelectric, and photocatalytic properties under biaxial strain were thoroughly examined by means of DFT simulations. The p-CGeP_4_ monolayer is identified as an indirect wide band gap semiconductor (*E*_gap_ = 3.20 eV) with dynamic stability, evidenced by the absence of imaginary phonon modes and remarkable thermal stability up to approximately 2050 K. The mechanical analysis confirms the material stability, with Young modulus ranging between 77.58 and 100.23 N/m, Poisson ratio from 0.03 to 0.26, and shear modulus from 31.01 to 48.55 N/m. Notably, p-CGeP4 exhibits significant direct (*e*_31_ = −11.27 and *e*_36_ = −5.34 × 10^–10^ C/m^2^) and converse (*d*_31_ = −18.52 and *d*_36_ = −13.18 pm/V) piezoelectric coefficients, surpassing other pentagon-based structures and indicating strong potential for applications in energy devices.

The monolayer can be homogeneously compressed or stretched up to −14% and +14% strain, respectively. Mechanical deformation significantly tunes its electronic structure with the band gap increasing to 3.31 eV under 4% tensile strain and decreasing to 1.97 eV at maximum stretching. In comparison, compressive strain reduces the band gap to 2.65 eV at −8% and further to 0.97 eV. Band edge alignment analysis shows that strongly basic conditions offer the most promising band alignment for the new p-CGeP_4_ monolayer, with potential photocatalytic behavior across all tensile strain regimes and significant compression levels (ε = 0% to −8%). The p-CGeP_4_ shows promising material applications in nanoelectronics, energy devices, and materials engineering, offering new perspectives for photocatalytic devices under stress conditions.

## Supporting Information Available

MD simulation for stability at 300 K by 40 ps (MP4)
MD simulation of heating from 300 K up to rupture (MP4)
